# Patient, Caregiver, and Clinician Perspectives on the Time Burdens of Cancer Care

**DOI:** 10.1001/jamanetworkopen.2024.47649

**Published:** 2024-11-04

**Authors:** Arjun Gupta, Whitney V. Johnson, Nicole L. Henderson, Obafemi O. Ogunleye, Preethiya Sekar, Manju George, Allison Breininger, Michael Anne Kyle, Christopher M. Booth, Timothy P. Hanna, Gabrielle B. Rocque, Helen M. Parsons, Rachel I. Vogel, Anne H. Blaes

**Affiliations:** Division of Hematology, Oncology, and Transplantation, University of Minnesota, Minneapolis; Division of Hematology, Oncology, and Transplantation, University of Minnesota, Minneapolis; Division of Hematology and Oncology, University of Alabama at Birmingham; Division of Hematology, Oncology, and Transplantation, University of Minnesota, Minneapolis; Division of Hematology, Oncology, and Transplantation, University of Minnesota, Minneapolis; Paltown Development Foundation/COLONTOWN, Crownsville, Maryland; The Negative Space, Minneapolis, Minnesota; Department of Health Care Policy, Harvard Medical School, Boston, Massachusetts; Division of Cancer Care and Epidemiology, Queen’s Cancer Research Institute, Kingston, Ontario, Canada; Division of Cancer Care and Epidemiology, Queen’s Cancer Research Institute, Kingston, Ontario, Canada; Division of Hematology and Oncology, University of Alabama at Birmingham; Division of Hematology, Oncology, and Transplantation, University of Minnesota, Minneapolis; Division of Hematology, Oncology, and Transplantation, University of Minnesota, Minneapolis; Division of Hematology, Oncology, and Transplantation, University of Minnesota, Minneapolis

## Abstract

**IMPORTANCE:**

Cancer and its care impose significant time commitments on patients and care partners. The oncology community has only recently conceptualized these commitments and the associated burden as the “time toxicity” of cancer care. As the concept gains traction, there is a critical need to fundamentally understand the perspectives of multiple stakeholders on the time burdens of cancer care.

**OBJECTIVES:**

To explore time-consuming aspects of cancer care that were perceived as burdensome, identify the individuals most affected by time burdens of cancer care, and evaluate the consequences of these time burdens.

**DESIGN, SETTING, AND PARTICIPANTS:**

Participants in this qualitative analysis were recruited from a National Cancer Institute–designated cancer center in Minnesota, where semistructured qualitative interviews were conducted from February 1 to October 31, 2023. Purposive and criterion sampling methods were used to recruit patients (adults with advanced stage gastrointestinal cancer receiving systemic cancer-directed treatment), care partners (patient-identified informal [unpaid] partners), and clinicians (physicians, physician assistants, nurse practitioners, nurses, social workers, and schedulers). Data were analyzed from February 2023 to February 2024.

**MAIN OUTCOMES AND MEASURES:**

Thematic analysis was conducted with a hybrid (inductive and deductive methods) approach. Themes, subthemes, and illustrative quotations are presented.

**RESULTS:**

Interviews included 47 participants (16 patients [8 aged ≤60 years; 12 women (75.0%)], 15 care partners [12 aged ≤60 years; 9 women (60.0%)], and 16 clinicians [11 women (68.7%)]). A total of 31 subthemes were identified that were grouped into 5 themes. Theme 1 captured time burdens due to health care outside the home (eg, travel, parking, and waiting time), while theme 2 identified the often invisible tasks performed at home (eg, handling insurance and medical bills, receiving formal home-based care). Theme 3 explored how care partners are affected alongside patients (eg, burdens extending to the wider network of family, friends, and community) and theme 4 represented the consequences of time burdens (eg, demoralization, seemingly short visits turned into all-day affairs). Finally, theme 5 referenced positive time spent in clinical interactions and hope for change (eg, patients value meaningful care, the “time toxicity” label is a spark for change).

**CONCLUSIONS AND RELEVANCE:**

This qualitative analysis identifies key sources and effects of time toxicity, as well as the populations affected. The results of this study will guide the oncology community to map, measure, and address future time burdens.

## Introduction

Cancer and its care impose significant time commitments on patients and care partners.^1^ These commitments, along with their associated burden, have recently been conceptualized as the “time toxicity” of cancer care.^2^ Patients with advanced solid tumors spend approximately 20% to 30% of their days alive with health care contact.^3-8^ The small survival benefits associated with some treatments may be offset by their time toxicity,^9^ and increased time toxicity is associated with worse quality of life.^10^

Although this recent focus on time toxicity is encouraging,^11^ the field is unfortunately limited by a small amount of foundational work to understand the sources, populations affected, and consequences of time toxicity. Prior work on financial toxicity has noted several sources of costs, how subjective experiences vary alongside objective costs, and downstream consequences.^12,13^ Similar concepts have not yet been mapped out for time toxicity. A complicating factor with time is that, while we can aspire to decrease financial and logistic costs to near zero (eg, prefer to pay $0 compared with $5000, and to completely avoid the frustration of an incorrect medical bill), some time spent on cancer care is necessary and value added. Identifying what components of care are perceived as value added from a time perspective by patients and care partners, vs what components are not, is critical to advancing the field. To get a 360° view of time toxicity, it is critical to also understand clinicians’ perspectives on patients’ time. Clinician engagement is critical to the success of eventual interventions to address time toxicity in cancer care.

Time is important to everyone; however, time toxicity is especially relevant when patients pursue time-intensive treatments in the context of limited survival. One such scenario is patients with advanced stage gastrointestinal cancer. Globally, gastrointestinal cancers account for one-fourth of new diagnoses and one-third of cancer-related deaths.^14,15^ Gastrointestinal cancers affect a broad population (across age, gender, and sex) and capture a multitude of clinical scenarios while providing a relatively homogenous background to contextualize experiences. The mean survival of patients with advanced gastrointestinal cancer is often less than 1 year.^4,5,16^ Throughout this period, patients often experience burdensome symptoms and complications.^17^ Thus, they form an ideal population with whom to study time toxicity. To build a foundational understanding of the sources, populations affected, and consequences of time toxicity, we elicited the perspectives of patients with advanced gastrointestinal cancer, their care partners, and oncology clinicians.

## Methods

### Participants, Recruitment, and Setting

We prospectively recruited participants from the University of Minnesota’s MHealth Fairview clinic. We used purposive and criterion sampling methods to recruit a demographically diverse population, striving for representation across cancer sites, age, gender, and socioeconomic status, and among clinicians, by role.^18,19^ We recruited 3 groups: (1) patients: adults with advanced gastrointestinal cancer, English speaking, receiving systemic treatment; (2) care partners: patient-identified informal (unpaid) partners, eligible even if a patient did not participate; and (3) oncology clinicians: advanced practice clinicians (physician assistants and nurse practitioners), nurses, physicians, social workers, and schedulers.

eMethods in Supplement 1 contains additional details. We conducted interviews in 2 phases: pilot (n = 9: 3 from each group; February 1 to March 31, 2023) and main (n = 38: May 1 to October 31, 2023). We collected participant characteristics using self-report (gender, race, etc) and the electronic medical record (eg, cancer site). We received approval from the University of Minnesota institutional review board and obtained verbal informed consent. We followed the Consolidated Criteria for Reporting Qualitative Research (COREQ) reporting guideline.^20^

### Interviews

An interdisciplinary team including patient and care partner advocates, researchers with expertise in qualitative methods and treatment burden, and oncologists developed the interview guide (eTable 1 in Supplement 1) after a literature review.^21^ We sampled 15 to 20 participants per group, aiming for saturation. A trained interviewer (O.O.O.) conducted 45-minute semistructured interviews and audio recorded and transcribed interviews. We refined the guide after piloting. The guide explored elements of care taking up time, how participants perceived time costs and burdens, experiences and perspectives, persons affected, and consequences.

### Analysis

We used thematic analysis, a type of grounded theory. This method supported a hybrid analytic approach, with some codes derived strictly from the data (inductive) and others identified in advance (deductive).^22-24^ We began constant comparative analysis from the start of interviews in February 2023 until February 2024 when coding was finalized. Two researchers (A.G. and O.O.O.) independently coded the data using NVivo, version 12 (Lumivero), compared codes, and merged them into a shared codebook. The codes and themes were continuously refined until consensus was reached.^25^

## Results

### Participant Characteristics

We interviewed 47 participants (16 patients, 15 care partners, and 16 clinicians) (characteristics in Table 1 and Table 2; eTable 2 in Supplement 1). Among the 16 patients, 8 (50.0%) were aged 61 years or older; 12 (75.0%) identified as women and 4 (25.0%) as men; 3 (18.7%) were Asian, 2 (12.5%) were Black, and 10 (62.5%) were White; and 8 (50.0%) had hepatopancreatobiliary cancers (Table 1). Among the 15 care partners, 12 (80.0%) were aged 60 years or younger; 9 (60.0%) identified as women and 6 (40.0%) as men; 2 (13%) were Asian, 2 (13.3%) were Black, and 11 (73.3%) were White; and 5 (33.3%) lived 15 minutes or more away from the patient for whom they provided care. The 16 clinician participants (11 women [68.7%] and 5 men [31.2%]) represented multiple perspectives (Table 2).

### Identified Themes

We identified 31 distinct subthemes grouped into 5 themes, presented here and in eTable 3 in Supplement 1, along with illustrative quotations.

#### Source of Time Burdens: Health Care Outside the Home (Theme 1)

Activities related to the pursuit, receipt, and recovery from care outside the home were major sources of time burdens. Participants acknowledged that components of care (eg, face-to-face time with clinicians) were essential, but also identified nonessential and burdensome aspects of care. These aspects included (1) time spent in travel, (2) parking, and (3) waiting, primarily related to attending ambulatory appointments. Time burdens were exacerbated for patients living in rural areas, and for patients unable to drive themselves to appointments.

Regardless of waits or delays, 3 subthemes described unique burdens: planned appointments, unplanned appointments, and overnight stays in a facility. Sometimes, even planned appointments, such as long infusions, and frequent multiple clinic visits were perceived as burdensome. Symptoms and complications resulted in unplanned visits to the clinic, urgent care, and emergency departments (EDs). There was added uncertainty in the timeline and outcomes of an ED visit (“It’s like a black hole of time, because you don’t know.”). The unfamiliarity of patients or care partners with non–oncology-specific domains (ED or other health systems) and vice versa was associated with more delays. These visits sometimes turned into overnight stays in facilities.

#### Source of Time Burdens: Often Invisible Tasks Performed at Home (Theme 2)

Although time at home is sometimes seen as universally good, participants illuminated extensive responsibilities that took up their time while at home, as described here in 9 subthemes. Participants described (1) performing logistic and administrative tasks—tasks associated with an opaque and inefficient health care system—as time burdensome. A clinician described these as “the enormous iceberg beneath the surface.” Other clinicians recalled themselves being “lost in a phone tree” with insurance companies, recognized patients had “many hoops to jump through,” and described such a task as a “rabbit hole.”

Key administrative burdens included (2) handling insurance and medical payments, (3) scheduling care, (4) communicating with the care team, and (5) legal and planning issues, such as estate planning. Tasks such as handling billing or legal issues, accessing benefits, and planning for end of life, took up more time than anticipated due to unfamiliarity and limited support. Scheduling care and communicating with the care team required playing “phone tag.”

In addition to time spent on administrative burdens, patients and care partners spent time on (6) educating themselves about the cancer or treatments, (7) direct medical care, (8) receiving formal home-based services, and (9) tending to and recovering from symptoms. A patient likened educating oneself about cancer care to “drinking from a firehose.”

Although home-based care alleviated travel and parking time costs, it was not a panacea. In one case, a patient opted for infusions in the clinic (vs at home) because they realized it would be more of a time commitment at home—the waiting and coordination for home care staff or services goes “under the radar.” Even when home, recovering from chemotherapy could take several days and even weeks.

#### Populations Affected: Care Partners Alongside Patients (Theme 3)

Although the aforementioned sources often affected care partners, participants additionally noted 4 specific features faced by care partners. First, care partners’ time was equally, if not more so, affected than patients’ time. Care partners’ time loss was described as “collateral damage.” Second, care partners were involved not only in 1 or 2 aspects of care but in every aspect of care. Many care partners shared that they attend nearly every medical appointment. Third, beyond attending to health care needs, care partners took over social responsibilities and tasks of daily living (eg, household tasks, financial responsibilities, and caregiving to other dependents). Fourth, there was a “domino effect” and “rippling effect” affecting not only the patient and their immediate (index) care partner, but also the entire network of family and friends who helped fill in gaps. This managing of multiple tasks was akin to “juggling,” and because the only “ball that wasn’t allowed to drop was the patient,” care partners and their extended networks had to adjust most, if not all, aspects of their lives.

#### Consequences of Time Burdens (Theme 4)

Time burdens were associated with wide-ranging consequences, covered in 8 subthemes. Three subthemes captured the broader psychosocial effect. (1) Cancer care could be all consuming—it became a “full-time job,” “a completely new vocation,” and “we do cancer, that’s what we do.” (2) They described a loss of control over their own life. This was likened to being “on a leash without any control,” “being unable to get traction” in daily life, and always feeling the need to be ready to pursue cancer care (“plan for the unplanned”). (3) Life revolved around cancer care—this was likened to an “extended [COVID-19] lockdown” that curtailed people’s life, and life had to be “spun” around cancer care. A clinician described this as “being on call—you may not get called in, but you need to be always available.”

Two additional subthemes covered more discrete effects: (4) having to take leaves of absence or retire from work, postpone travel, miss events such as weddings and holidays, and give up hobbies and joyful social activities; and (5) for ambulatory appointments, even supposedly “quick” clinic trips (eg, for drawing blood for laboratory testing) could take up several hours’ time, and was perceived as an entire day’s loss.

Frequent appointments served as (6) emotional reminders of illness. Even after visits were over, patients needed to “mentally decompress,” which took up more time. Logistic and administrative tasks, especially when opaque, were (7) demoralizing—after spending hours dealing with insurance companies, issues remained unresolved, and in some cases, patients “gave up” and ended up “just paying.” Last, (8) time burdens were linked with financial burdens—either due to decreased income and/or care costs.

#### Positive Interactions and Hope for Change (Theme 5)

This theme is an amalgamation of “positive” findings that emerged, covered in 4 subthemes. (1) One can think about time spent only negatively, but patients and care partners recognize that some cancer care is essential, and acknowledge the value of the time and efforts of the clinical team. They recognize that needs are unpredictable, and sometimes even “unscheduled” but necessary care can be rewarding. A care partner noted how the time spent driving to appointments brought the patient and them closer.

The remaining subthemes emerged from the clinicians’ perspectives. Some clinicians acknowledged that (2) oncology has traditionally not appreciated patients’ time loss (“we are disdainful of patients’ time”). They recognized the need for change, and to not do things as “ritual.” Clinicians noted how (3) time burdens “have been around forever,” but how the “label of time toxicity may be a lightbulb moment,” and a call to action. Last, reflecting on the time burdens for patients and care partners, (4) clinicians shared their own time burdens and how this was a barrier to fixing patients’ time toxicity.

## Discussion

In this qualitative study including perspectives of multiple stakeholders, we sought to develop a fundamental understanding of the time toxicity of cancer care. Participants recognized that some cancer care was necessary, and valued meaningful time spent receiving cancer care, but highlighted several sources of unnecessary time burdens, both while receiving care outside the home and when at home. In addition to patients, these burdens affected care partners and the extended network around them. These time burdens had extensive effects on patients and care partners. Encouragingly, clinicians acknowledged that oncology has not always respected patients’ time, and hoped the “time toxicity” label is a spark for change. Together, these results provide a foundational understanding of the time toxicity of cancer care.

Participants reported that attending frequent, uncoordinated, ambulatory appointments was often exceptionally burdensome, especially when accounting for travel, parking, and waiting times, as well as the burdens of scheduling and coordination. Most patients in this study lived more than 15 minutes from the primary treatment site, which was located in a dense, urban area, with paid parking garages and valet parking available. They noted that time and financial costs associated with parking were substantial.^26,27^ A previous study has demonstrated that the mean round trip walk time from a garage spot to the clinic entrance is 14 minutes.^28^ Parking costs reported in the current study ($12/day) were similar to national patterns.^29^ Modern oncology care is multidisciplinary and delivered largely in the outpatient setting, requiring extensive communication between teams and the patient or care partner. In quantitative work among a similar population, patients spent approximately 1 in 4 days with health care contact—a major source was ambulatory appointments.^5^ Similar patterns are seen for patients with advanced cancer receiving care at Veterans Affairs facilities, in Canada, and during clinical trials.^4,6,9,30^ Ambulatory appointments for patients with cancer are often uncoordinated, with services such as clinician visits, bloodwork, scans, procedures, and infusions not scheduled on the same day.^31^ Participants reported that seemingly short appointments turned into all-day affairs. This likely reflects the serial nature of oncology workflow and systemic inefficiencies, and mirrors prior qualitative work among persons with cancer, where a care partner commented that “You can pretty much plan a whole day [at the cancer center].”^32^ Using real-time location system and geolocation data, a study has demonstrated that the median round-trip time for patients seeking ambulatory cancer care in a single day is 197 minutes (IQR, 143-287 minutes).^28^ Together, these data support “contact days” as a patient-centered measure of time toxicity, as is currently being used in the oncology community.^4,6,31,33-36^ Possible solutions include optimizing the necessity, coordination, frequency, modality, and length of visits; better supporting patients and care partners with logistics^12,37,38^; and proactively informing them about expected experiences. Telehealth, when appropriate, can also potentially mitigate time toxicity. Our ongoing work focuses on evaluating solutions to address time toxicity, with specific attention to ensuring this does not worsen disparities for vulnerable individuals and communities.^39^

Three salient points about time burdens at home merit further discussion. First, participants frequently highlighted the burden of logistic and administrative tasks—these were unexpected and demoralizing (also due to the opaque processes, lack of support from the care team, and eventual failure). Work in the non–oncology-specific context has highlighted that almost 60% of patients engage in tasks such as obtaining prior authorizations or resolving billing problems.^40^ Given the costly and complicated management paradigms in oncology, it is plausible that these burdens are even more common for patients with cancer.^41^ We observed all 3 previously described categories of administrative burdens: learning (eg, learning about a program, determining eligibility), compliance (eg, tasks such as paperwork and telephone calls), and psychological costs (eg, stress, loss of autonomy).^42^ Second, home-based care was not universally perceived as saving time relative to receiving the same care in a clinic. A patient described how in a clinic they had more clarity to show up at a specific time, vs waiting for home care staff. As health systems and payers push care into homes, we should be mindful of patient and care partner burdens, capacity, and priorities before universally applauding this as time saving.^43^ Third, we noted how care partners’ lives could be completely consumed with cancer care, and how this time burden spilled over to the larger group of friends, family, and community. This does not diminish the burdens faced by the index care partner, but highlights that even capturing the burdens of the patient-care partner dyad may not fully capture the time burden of cancer care.

In line with prior work, we found that frontline clinicians across roles recognized that oncology had a time toxicity problem.^32^ Although clinicians’ recognition of time burdens and the prevalence and severity of time burdens may appear discordant, this reflects the fact that clinicians do not deliver care in a vacuum—cancer care is complex, and important stakeholders may have different priorities than minimizing patient time burden. In fact, in many cases, inefficiency is profitable for certain stakeholders, contributing to increased logistic and administrative burdens. We believe that clinicians in this study were sensitized to patient time burdens due to their own experiences handling logistic and administrative burdens while trying to ensure the best possible cancer care. We did not probe or specifically ask clinicians about their own time burdens, but they reflected spontaneously how their already precious time in clinic with the patient was cut into by non–value-added administrative tasks. This impeded their ability to think, empathize, and provide the best possible care.

### Limitations

This study has some limitations. First, these findings may not be representative of viewpoints of the entire community of people with lived or professional experience of cancer. We included persons affected by gastrointestinal cancers only, treated primarily at an urban center with travel times generally less than 30 minutes. Patients residing in rural areas, non–English-speaking patients, or patients with cancers requiring more intensive treatment (eg, hematologic cancers) may have different perspectives. Objective time costs may translate into different subjective time burdens, with the most vulnerable individuals and populations at greatest risk of time toxicity.^39^ We made proactive efforts to ensure diversity with respect to age, gender, race, and socioeconomic status, with varying clinical and treatment characteristics. Second, by requiring patients to have undergone cancer-directed therapy, we may have excluded perspectives from patients who opted not to receive cancer-directed therapy due to the associated time burdens. Third, participants in this study did not report forgoing care related to time burdens; this may represent a true absence of an association, or the fact that all participants were insured and already receiving cancer care.

## Conclusions

This qualitative study provides a foundational understanding of the sources, populations affected, and consequences of time toxicity of cancer care. This comprehensive investigation will aid efforts to map, measure, and intervene on time toxicity.

## Supplementary Material

Supplemental 2 Data Sharing

Supplemental 1 **eTable 1.** Sample Interview Guide for Patients for the Exploratory Portion of the Interview
**eTable 2.** Detailed Participant Characteristics
**eTable 3.** Themes, Subthemes, and Illustrative Quotations

## Figures and Tables

**Table 1.** Participant Characteristics for Patients and Care Partners

Characteristic	No. (%)	
	Patients (n = 16)	Care partners (n = 15)
Age, y		
18-30	0	2 (13.3)
31-45	2 (12.5)	6 (40.0)
46-60	6 (37.5)	4 (26.7)
61-75	7 (43.8)	3 (20.0)
≥76	1 (6.2)	0
Self-identified gender		
Women	12 (75.0)	9 (60.0)
Men	4 (25.0)	6 (40.0)
Self-identified race		
Asian	3 (18.7)	2 (13.3)
Black	2 (12.5)	2 (13.3)
White	10 (62.5)	11 (73.3)
Not reported^a^	1 (6.2)	0
Primary cancer site^b^		
Colorectal	6 (37.5)	6 (40.0)
Gastroesophageal	2 (12.5)	2 (13.3)
Hepatobiliary	5 (31.2)	4 (26.7)
Pancreatic	3 (18.7)	3 (20.0)

a One patient participant opted not to report race.

b For care partners, for their corresponding loved one (patient).

**Table 2.** Participant Characteristics for Clinicians

Characteristic	Clinicians (n = 16), No. (%)
Self-identified gender	
Women	11 (68.7)
Men	5 (31.2)
Credentials	
Advanced practice clinician^a^	2 (12.5)
Clinic scheduler	1 (6.2)
Physician	7 (43.8)
Nurse	4 (25.0)
Social worker	2 (12.5)
Years of experience	
<5	3 (18.7)
5-10	5 (31.2)
11-20	5 (31.2)
>20	3 (18.7)

a Physician assistants and nurse practitioners.

## Data Availability

See Supplement 2.

## Key Points

### Question

What are the sources of time burdens for patients with cancer, who is affected by these time burdens, and what are the consequences of these time burdens?

### Findings

In this qualitative analysis of patients, informal care partners, and clinicians, participants highlighted the diverse sources of time burdens, how these time burdens affected care partners alongside patients, and how the time burdens extended to the wider network around them. Time burdens had outcomes ranging from causing psychosocial distress, and “seemingly short” ambulatory appointments turning into all-day affairs.

### Meaning

These findings will guide the oncology community to map, measure, and address time burdens for persons affected by cancer.
