# Supplementary material for: Patient, Caregiver, and Clinician Perspectives on the Time Burdens of Cancer Care
Source: JAMA Netw Open. Author manuscript; Available in PMC 2025 Apr 29. (PMC12040224; doi:10.1001/jamanetworkopen.2024.47649)
Supplement: Supplemental 2 Data Sharing [file NIHMS2072801-supplement-Supplemental_2_Data_Sharing.pdf]

Data Sharing Statement

Gupta. Patient, Caregiver, and Clinician Perspectives on the Time Burdens of Cancer Care. *JAMA Netw Open*. Published November 27, 2024. doi:10.1001/jamanetworkopen.2024.47649

Data
Data available: No

Additional Information
Explanation for why data not available: The data underlying this article cannot be shared out of privacy for the individuals that participated in the study. The qualitative nature of the interviews and specific experiences of patients and care partners are personal, and even removing identifying information from individual transcripts may still breach confidentiality.
