# Supplementary material for: Patient, Caregiver, and Clinician Perspectives on the Time Burdens of Cancer Care
Source: JAMA Netw Open. Author manuscript; Available in PMC 2025 Apr 29. (PMC12040224; doi:10.1001/jamanetworkopen.2024.47649)
Supplement: Supplemental 1 — eTable 1. Sample Interview Guide for Patients for the Exploratory Portion of the Interview eTable 2. Detailed Participant Characteristics eTable 3. Themes, Subthemes, and Illustrative Quotations [file NIHMS2072801-supplement-Supplemental_1.pdf]

Supplementary Online Content

Gupta A, Johnson WV, Henderson NL, et al. Patient, caregiver, and clinician perspectives on the time burdens of cancer care. *JAMA Netw Open.* 2024;7(11):e2447649. doi:10.1001/jamanetworkopen.2024.47649

eMethods.
eTable 1. Sample Interview Guide for Patients for the Exploratory Portion of the Interview
eTable 2. Detailed Participant Characteristics
eTable 3. Themes, Subthemes, and Illustrative Quotations

This supplementary material has been provided by the authors to give readers additional information about their work.

© 2024 Gupta A et al. *JAMA Network Open.*

eMethods.

We identified potentially eligible patients and care partners during clinic visits; they were approached by a study coordinator unrelated to the treatment team. Potential clinicians were approached by the study coordinators in clinic.

Patient and care partner participants were offered $25 gift cards.

Once the interviewer perceived that data saturation was reached, we concluded scheduling additional interviews and moved to formal data analysis.

Interrater reliability was assessed using NVivo, with a >0.7 Cohen's kappa level of agreement considered acceptable. We mapped the relationships between subthemes and themes.

McHugh ML. Interrater reliability: the kappa statistic. Biochem Med (Zagreb). 2012;22(3):276-282.

Novak JD. Concept Mapping: A Useful Tool for Science Education. In. Vol 27(10). Journal of Research in Science Teaching:937-949.

Kane M, Trochim WM. Concept mapping for planning and evaluation. In: Sage Publications, Inc; 2007.

© 2024 Gupta A et al. *JAMA Network Open.*

eTable 1. Sample Interview Guide for Patients for the Exploratory Portion of the Interview

Introduction
Hello, my name is [______], and I am a [JOB TITLE/ROLE] from the University of Minnesota. I'm currently working on a project studying the time burdens of cancer treatment and their effects on patients and their families. While there are some overlaps, the cancer treatment experience is different for everyone, so we're talking with a number of different patients about what the process was like for them.

The interview should take about 30-45 minutes and as we go through the questions, please know that your participation is completely voluntary, and you may choose not to answer to end the interview at any time.

If it's okay with you, I would like to record our interview, so that I can focus on our conversation now but still compare your experiences to other people that I talk to. The only people who have access to the recording will be our trained research staff and if we use the information you share in presentations or publications, it will be anonymous. Is it okay if I record?

Part 1: Exploratory Questions

1. We are interested in exploring the entire process of your experience with cancer care. What activities took the most time, starting from when you were first diagnosed, or perhaps even before that?
   How have things changed over time?
2. Among the different things mentioned, which ones have been the most time burdensome for you?
   a. What makes them burdensome?
3. How have these activities that take up time affected your life?
4. We are also curious about how the time of cancer care has affected your loved ones, such as friends and family who support you.
5. Is there anything else you would like to add? Anything you feel was missed in this discussion?

End of Interview

© 2024 Gupta A et al. *JAMA Network Open.*

eTable 2. Detailed Participant Characteristics

Patients (n=16)	
**Characteristic**	**Number (percentage)**
**Cancer stage**	
Locally advanced	8 (50.0)
Metastatic	8 (50.0)
**Treatments in addition to systemic therapy**	
Radiation alone	0 (0.0)
Surgery alone	6 (37.5)
Radiation and surgery	4 (25.0)
None	6 (37.5)
**Lines of systemic treatment (including current)**	
One	3 (18.8)
Two	7 (42.8)
Three	3 (18.8)
Four or more	3 (18.8)
**Living situation**	
Living alone	5 (31.3)
Living with adult care partner	10 (62.6)
Living with adults care partner and other dependents	1 (6.2)
**Care partner interviewed**	
Yes	13 (72.2)
No	3 (18.8)
**Residence**	
Rural	13 (72.2)
Urban	3 (18.8)
**Work status**	
Full time	11 (68.8)
Part time	4 (25.0)
Unemployed/ leave of absence	0 (0.0)
Retired	5 (31.3)
**Primary insurer**	
Medicaid	1 (6.2)
Medicare	2 (12.5)
Private	8 (50.0)
Private + Medicare	5 (31.3)
**Education level**	
High school diploma	1 (6.2)
Associate's degree	5 (31.3)
Bachelor's degree	6 (37.5)
Advanced degree	4 (25.0)
**Household Income (dollars per year)**	
15,000 to 25,000	1 (6.2)
25,000 to 50,000	5 (31.3)
50,000 to 100,000	6 (37.5)
More than 100,00	4 (25.0)
**Travel time to cancer center (minutes)**	
Less than 15 minutes	5 (31.3)
15 to 30 minutes	8 (50.0)
31 to 60 minutes	3 (18.8)

© 2024 Gupta A et al. *JAMA Network Open.*

Care partners (n=15)	
**Characteristic**	**Number (percentage)**
**Relation to patient**	
Children/children-in-law	6 (40.0)
Friend	2 (13.3)
Parent	2 (13.3)
Partner/spouse	4 (26.7)
Sibling	1 (6.6)
Driving distance from patient	
Lives with patient	7 (46.7)
Less than 15 minutes	3 (20.0)
15 to 30 minutes	3 (20.0)
31 to 60 minutes	2 (13.3)
Caring for other individuals	
Yes, children	3 (20.0)
Yes, another adult	2 (13.3)
No	10 (66.7)
**Education level**	
High school diploma	1 (6.6)
Associate's degree	2 (13.3)
Bachelor's degree	9 (60.0)
Advanced degree	3 (20.0)
**Work status**	
Full time	7 (46.7)
Part time	3 (20.0)
Unemployed/ leave of absence	3 (20.0)
Retired	2 (13.3)
**Caregiver has own health issues (self-reported)**	
Yes	3 (20.0)
No	12 (80.0)
**Time being a caregiver**	
Less than 1 year	7 (46.7)
1 to 2 years	5 (33.3)
2 to 4 years	2 (13.3)
More than 4 years	1 (6.6)

© 2024 Gupta A et al. *JAMA Network Open.*

eTable 3. Themes, Subthemes, and Illustrative Quotations

**Theme 1: Source: Healthcare outside the home**	
**Subthemes**	**Illustrative quotations**
**Travel time**	*''My wife and I made over 100 drives in two years from my house, all the way [to the clinic,] again, over an hour and a half in each direction. The drive there, the drive back. That's the burden. And during that time, that was painful. You're in pain, and then you have to get in a car and drive. All those bumps.''* (Patient)<br><br>*''So from my home to my mother's home it's about a 40 minute drive each way. So for example, if I'm going to take her to clinic, I leave my home, go to her home, that's 40 minutes there. And then I take her out to clinic and that's another 30ish minutes. And I live 10-15 minutes away from clinic.''* (Care Partner)
**Parking time**	*''You have to realize just going to [the urban clinic] there is a parking problem. It takes ages.''* (Patient)<br><br>*''I wish we had better parking ramps that if you're going to be there X amount of days. We used valet as well. It was faster, but still took time.''* (Care Partner)
**Wait time**	*''My mom would check in, have her labs in a timely fashion and then we would wait because the medication that they needed for her infusion was not ready.''* (Care Partner)<br><br>*''There's a lot of it spent waiting on labs. A lot of waiting. Just stuck waiting in the rooms on lab results before they can see if they're gonna get treatment for the day. A lot of patients complain about how long it takes.''* (Clinician)
**Attending planned clinic appointments and picking up medications**	*''Three out of five days a week, we were in infusion for four hours and that's a big toll. A big big toll.''* (Care Partner)<br><br>*''I think when patients look at [the schedule] and they go, why couldn't they just do this all on one day? I think that that would probably be if it was me, it would be my biggest frustration and it's definitely a frustration I hear from patients where, they, they constantly talk about multiple trips.''* (Clinician)
**Urgent care and Emergency department visits (unplanned visits)**	*''When you go to the emergency room you have no idea when you're going to get to leave, how long you're going to sit in that waiting room. It just turns into an ordeal. It's like a black hole of time, because you don't know.''* (Patient)<br><br>*''And she was treated through [health system], so we wanted to go to [health system's] hospital but we called an ambulance, they said, there are big backups there, we're worried she could wait a long time, so then we went to a different ER, and then that problem became even bigger. Nothing was integrated and suddenly, we're like, Well, hey, wait a minute, what is going on?''* (Care Partner)
**Hospitalizations and facility-based care**	*''I've got sepsis twice during my chemotherapy. So, I ended up going into the hospital for a week and they're in there bombing me with antibiotics. And then. I recovered from that. But it took five days or six days away from me. I was so surprised.''* (Patient)

© 2024 Gupta A et al. *JAMA Network Open.*

	''Now with all the hospital stays, I've spent a lot of time and transportation and being with her and so it really impacts me and my family.'' (Care Partner)
**Theme 2: Source: Often invisible tasks performed at home**	
**Subthemes**	**Illustrative quotations**
**Logistic and administrative tasks related to cancer/ treatment**	*''So, even just thinking a little bit about time spent. It's an enormous iceberg that is beneath the surface. What we see in clinic is just the tip. There's all this other time that's spent.''* (Clinician)<br><br>*''I think some of the most burdensome are the phone calls. There are so many different pieces. So, just like calling us to get everything scheduled, maybe it's insurance, calling for financial help. It's never really easy. And then someone is not available. It's playing phone tag.''* (Clinician)
**Handling insurance and medical bills**	*''My cancer surgery was miscoded as a weight loss surgery since they removed part of my stomach. I got a 60,000 dollar bill for that. I've been on the phone for, I don't even know, so long, on the phone with the insurance company, the hospital.''* (Patient)<br><br>*''And you just have to say, well, your insurance company is not approving it, we just have to supply more information and see what they say and I think that they when they realize that now they're down this rabbit hole of calling their insurance companies and they speak to three different people and no one you know, no one's on the same page.''* (Clinician)
**Advance care planning and other legal issues**	*''So for instance, unfortunately, so [patient] did not have, she did not have a will. So we kind of had to start trying to like sort through bank account assets, retire, retirement accounts, Social Security, disability. Were kind of some of like, the different applications we had for that sort of thing. We also had to get a health care directive or her I had to fill out a long-term disability application assistance and financial aid application. So those were kind of different sorts of just non-medical related things.''* (Care Partner)
**Scheduling care**	*''The scheduling was really annoying. And there were times too when they did schedule them, but they scheduled them at the wrong location. So, I had to be constantly like checking somebody's out somebody else's work. And that was exhausting.''* (Patient)<br><br>*''I get assailed with scheduling emails, reminders, eCheck-ins. I am coming to the appointment, okay? I don't need all this. It's already taking up my life.''* (Patient)
**Communicating with the care team**	*''I've called and I've had to wait, because oh, they don't know, they have to ask someone else''.* (Care Partner)<br><br>*''And even just communicating with her doctors too. She doesn't use technology. I have to sit down and message her providers or someone from her care team and then you know, take care of that back and forth. It's a lot.''* (Care Partner)
**Learning about the disease and treatments**	*''Well, everybody says when you're newly diagnosed, it's drinking from a firehose, and I spent a lot of time just trying to get up to speed on the fundamentals of the disease and trying to figure out everything that was happening and going to happen to me.'' (Patient)*

© 2024 Gupta A et al. *JAMA Network Open.*

**Medical care and medication management**	*‘’So yes, I've kind of broken it up into medical responsibilities for my [relationship]. It was an hour in the morning. And then we had medications that was every two to four hours, we were distributing many medications. She had a feeding tube that required flushing within the tube every four hours, and then we would have another additional hour in the evening. And then the hour in the morning and an the hour in the evening. There was kind of a, like a lot of different things that we would do within that time.’’* (Care Partner)
**Formal home-based care**	*‘’So, instead of going to the clinic, I could do everything at home, and my provider at the time, was giddy at that prospect. She was like, "This would be an amazing thing for you, you don't have to go to the clinic." But then when I started digging into how this would work, I realized the time burdens would be greater by doing it at home. And that has happened before where I've just spent the whole day at home waiting for home care. It's just one more example of something that sometimes goes under the radar.’’* (Patient)<br><br>*‘’Her care team also set up for home infusions to actually go out to my mom's home to de-access her because she has an infusion pump. So she'll go get an infusion in the clinic and then she's hooked up to an infusion pump that has to run for another 46 hours. Then we need to coordinate the home visit. Those 3 days are spent all around the chemo.’’* (Care Partner)
**Symptoms and recovery**	*’You can feel good the first day [after chemo] but the next day all the other medications, I think really often all the little steroids that they gave you during the chemo, is wearing off and then you feel like shit for the next two weeks. I think I didn't realize how much it would take me out of things for that like those two weeks.’’* (Patient)<br><br>*‘’So they're having to come get their treatment and essentially be in bed the rest of the day, sometimes for several days, that week when they're getting treatment and so on. Tons and tons of examples of that.’’* (Clinician)
**Theme 3: Populations impacted: Care partners alongside patients**	
**Subthemes**	**Illustrative quotations**
**Care partners time was equally if not more impacted than patients**	*‘’Anything that takes time for me, is almost an equal burden on her. It is an equal burden if not more sometimes.’’* (Patient)<br><br>*‘’At the end of the day, that I'm just so tired of constantly adulting because I feel like I'm doing the work for three adults.’’* (Care Partner)<br><br>*’’Every patient who is experiencing side effects and struggling at home or needs to come into the ER, hospital - that is more often than not affecting the network around them, you know, there is some collateral damage to the spouse or sibling or child of the patient.’’* (Clinician)
**Burdens related to direct medical care**	*‘’I have been to probably 90% of the appointments. Somebody's got to always be there to help her get in and out of the van, to appointments, to the clinic, to the hospital.’’* (Care Partner)<br><br>*’Like, if you look at her patient portal, the contact info is actually mine and that's because if they called her, she doesn't answer her phone. So I spend a lot of time just scheduling her appointments, letting her know, coordinating.’’* (Care Partner)
**Taking over social responsibilities and daily living**	*‘’Instead of spending time doing things [care partners] want to do, they're helping us do things we have to do. You can't do the little things, shoveling snow, mowing lawn, house maintenance. That would be where my father primarily has picked up everything. You name it.’’ (Patient)*

© 2024 Gupta A et al. *JAMA Network Open.*

	''There's also finances, managing all of the bills paying for. So, just managing all of that. I mean, last week, I was on the phone for hours trying to get a housing inspection and hiring an electrician to do some updating for a new insurance policy for her home.'' (Care Partner)
**Extending to wider network of family, friends, and community**	*''It's a domino effect. It just goes so much beyond, even me, it goes beyond me to all the people supporting.''* (Care Partner)<br><br>*''[We were] juggling and the only ball that wasn't dropping was [patient] because obviously she needed us then. So, yes, there was many times where my husband had to pick up some of my slack where --oh, the one day I forgot [my son] is done at work at one, not three. So, then it was like my husband had to go pick him up as everything was kind of on the backburner.''* (Care partner)
**Theme 4: Consequences of cancer care associated time burdens**	
**Subthemes**	**Illustrative quotations**
**Cancer care became all consuming**	*''I think now if we had to start all over again, I think it's a better expectation, but that first one was, Oh, my God, this is our life. This is our full-time job. This is all we do. We do cancer, that's what we do.''* (Care Partner)<br><br>*''One of my earliest thoughts was, I have a completely new vocation, which is to be a patient. I've got two teams and am a parent and work. And this feels like a complete one. And I, when I say vocation, like a complete vocational change, it's not like my, just my day job has changed, like my 24/7 job has changed.''* (Patient)
**Loss of control**	*''And the time piece is like a constant through all the cancer. It's like I am always on a leash. I really don't have any control.''* (Patient)<br><br>*''I don't feel like I can't get traction with anything I do, because a bomb could be thrown in at any moment. Or there's another appointment that I got to take and go like, I feel like I have to be able to drop everything at any moment.''* (Care Partner)
**Life needs to be planned around cancer care**	*''I think it's changed a lot in that it's almost like plans need to be made around appointments, rather than I make plans and schedule appointments around my plans. We're coming off of two-three years of lockdown as well. So it kind of feels like we're still in extended lockdown with cancer and can't really do much.''* (Care Partner)<br><br>*''So, in oncology, the cancer is perhaps not the most challenging piece, it's life that has to spin around it.''* (Clinician)
**Missing important life events and work, giving up hobbies**	*''It's one thing to just say, I can't go to that dinner or I can't take that one trip, but It has literally been everything from work to hobbies. I used to be very active in a book club, going out with friends regularly and being able to go to the gym in a reliable fashion. I mean, one-by-one all of these things got taken off the table.''* (Care Partner)<br><br>*''I know patients that have missed graduations or family reunions because of their treatment. They had to give up certain life events within their families. I hear that all the time.''* (Clinician)
**Seemingly short visits turned into all-day affairs**	*''Even for a 30 minute infusion, you'll be there, like half a day or a whole day, just getting it done. It kind of just robs your whole day. You make compromises. You have to reschedule other things, you know''* (Patient)

© 2024 Gupta A et al. *JAMA Network Open.*

	''And so, you know, some of the times appointments for one might be at 8:30 or 9:00 in the morning, another one might be at two in the afternoon, so, you know, it quickly turned into an all-day event because it wasn't worth driving back home in between those appointments.'' (Care Partner)
**Clinic visits and associated tasks are emotional reminders of illness**	*''I'm just trying to think through it all because again, there's an emotional part. There's just an emotional mental toll to the visits. And I actually don't know how much time it takes up, because you know, its beyond the visits.''* (Care Partner)<br><br>*''In my case, when I would have infusion days at the clinic, I know, at least a couple hours later, I need to decompress from that. Not just the physical component when there. And the mental component as well later.''* (Patient)
**Logistic and administrative burdens are demoralizing**	*'' We spent hours on the phone with no success trying to get the bill settled. What are we supposed to do? The social worker tried to help but everyone has been powerless. I needed to pick new insurance as well. It's a lot, we didn't know what to do.''* (Patient)<br><br>*'''The EOB's [explanation of benefits] don't line up with the provider bills even remotely. And so then it becomes extremely confusing. Like, what am I supposed to actually pay? And there are endless bills coming in because the treatment is constant. So, it's very difficult to figure out, wait, how much are we actually supposed to pay? And if we were to contest, if they said no, that actually we shouldn't pay, there's no ability to tell the hospital like, Hey, we're going to pay 90% of this bill, but this 10%, that never happened, I think that's a mistake. And its incredibly frustrating and time consuming, and was unexpected. We just end up paying.''* (Care Partner)
**Financial burdens linked to time burdens**	*''$12 to park — its $13 now— $13 to park at the clinic, and then come back and have to park again. That's another $13. And then you know, you have to come back the following week for labs. I mean, it adds up quickly.''* (Clinician<br><br>*''Well, for two weeks, I come every day at 7am and I'm done at three. And I do that for two weeks straight. The fact that you live two hours away, is not factored. The fact that I also work full time is not factored in. The fact that I'm caring for someone else at home is not factored in. And you start to see the fact that there's distance of two hour drive, but there's tolls, and those tolls are six bucks each way, in total. So, that's $12 a day. It's 15 bucks to park the car. And you and I both have to eat while we're here. And so now I've got financial, I've got travel, I've got childcare, I've got loss of employment,.''* (Clinician)

© 2024 Gupta A et al. *JAMA Network Open*.

Theme 5: Positive interactions and hope for change	
**Subthemes**	**Illustrative quotations**
**Patients value meaningful care**	*''So, Dr. [name], because of the wonderful man that he is, called and scheduled and did an urgent visit the next day, and just gave her permission to go ahead and sign up for hospice. She needed to feel comfortable with that decision. And Dr. [name] was able to give that to her. So, while that was an unscheduled visit, and we had to run around, it is exactly what we needed.''* (Care Partner)<br><br>*''For me, my experience has been really, really good. When I have had to be scheduled for any of my appointments, I have been called by the schedulers themselves, who said, 'This is what you need; this is when you need it; this is where you need to go,' and it's been great. [Scheduler name] in particular, we speak so often, its almost we are friends. I haven't had to make phone calls and then to wait on hold or anything, so, for me, I've had nothing but good luck with it.''* (Patient)
**Clinicians recognize oncology has a time toxicity problem**	*''Patients having to walk through this incredibly long, circuitous way to check in, do labs as ritual—we are indifferent to, and even disdainful of a patient's time. We should demand change.''* (Clinician)<br><br>*'I can't think of a more time-consuming health care specialty, even for sort of similar, incurable diseases. The things that we do, and we ask for are really quite immense. It is sad. I think oncology should learn from other disciplines. We are in our own bubble.''* (Clinician)
**The ''time toxicity'' label is a spark for change**	*''I really think that the biggest issue with ''time toxicity'' is getting the word out. People aren't going to factor it in if they don't have the word for it. That's a lightbulb moment of like yeah, of course that makes great sense. I've been around 25 years doing this. It was always obvious, but it's not until this term came that it really struck me.''* (Clinician)<br><br>*''Patients decide not to take a trip or you know, it's a regular occurrence that people decide to skip work for X days after chemo, but this is not something you know, we capture in clinical trials. People don't go to the ER because they missed a vacation. You know, it's just not, it's not a formally recognized adverse event. Maybe it should be. Maybe we need to capture these subclinical events as ''time toxicity''. Instead of them just being kind of in the background of everything that happens.''* (Clinician)
**Addressing clinicians' time burdens may help patients' time burdens**	*''You know, we have to do, we have to kind of spend a lot of time doing peer to peer, there's filling forms for insurance. And, the patient also has to do their part. But I guess it takes up so much of my time. There's not much time to work on [time burdens faced by patients].''* (Clinician)<br><br>*''I don't think that they [patients] realize that there's a lot that goes into ensuring that they don't end up with $100,000 bill. We do so much in the background. And I get it, it must be so stressful for them to not have an answer, why their care is delayed. I would call and explain things to them, but I am on the phone with the insurance company also.''* (Clinician)

© 2024 Gupta A et al. *JAMA Network Open*.
